# Clinical, Dermatoscopic, and Histological Findings in a Diagnosis of Pityriasis Lichenoides

**DOI:** 10.7759/cureus.8725

**Published:** 2020-06-20

**Authors:** Dillon D Clarey, Scott R Lauer, Ryan M Trowbridge

**Affiliations:** 1 Dermatology, University of Nebraska Medical Center, Omaha, USA; 2 Pathology, University of Nebraska Medical Center, Omaha, USA

**Keywords:** pityriasis lichenoides, dermoscopy, histopathology

## Abstract

Pityriasis lichenoides et varioliformis acuta (PLEVA) is a rare cutaneous eruption of erythematous macules and papules distributed over the flexural surfaces and the trunk. Histopathologic analysis is useful in diagnosis, and dermoscopic findings have been described in several small case series. We present a case of a mid-20s female who was diagnosed with PLEVA based on clinical and histopathological findings, and we also demonstrate a unique dermoscopic finding. Additionally, we review the current literature detailing dermoscopy findings with associated histopathology in PLEVA and pityriasis lichenoides chronica (PLC).

## Introduction

Pityriasis lichenoides is a dermatologic diagnosis consisting of three main forms: pityriasis lichenoides et varioliformis acuta (PLEVA), pityriasis lichenoides chronica (PLC), and febrile ulceronecrotic Mucha-Habermann disease (FUMHD). Histologically, PLEVA is characterized by focal changes to the epidermis and dermis [1]. A vacuolar interface dermatitis with a perivascular lymphocytic infiltrate involving the superficial vascular plexus that extends into the deep reticular dermis in a wedge-shaped pattern can be seen. Erythrocyte and lymphocyte extravasation into the epidermis and epidermal necrosis may also been seen [1,2]. Dermoscopy has proven most beneficial in the diagnosis of pigmented lesions, although several reports have described characteristic dermoscopy findings in both PLEVA and PLC [3-7]. Here, we present a case of PLEVA diagnosed with unique dermatoscopic findings.

## Case presentation

An Asian female in her mid-20s with no significant past medical history presented with a one-month history of an erythematous and pruritic rash preceded by an upper respiratory infection; anti-streptolysin O (ASO), throat culture, and monospot tests were not performed at that time. The rash initially started on her forearms and then spread to her upper arms, back, and stomach. She reported that the rash was pruritic, waxed and waned, and worsened at night. She denied any new medications, foods, soaps, detergents, or cohabitating with other itchy individuals. She had previously been treated unsuccessfully by primary care with oral methylprednisolone, hydrocortisone 1% cream, and permethrin.

**Figure 1:**
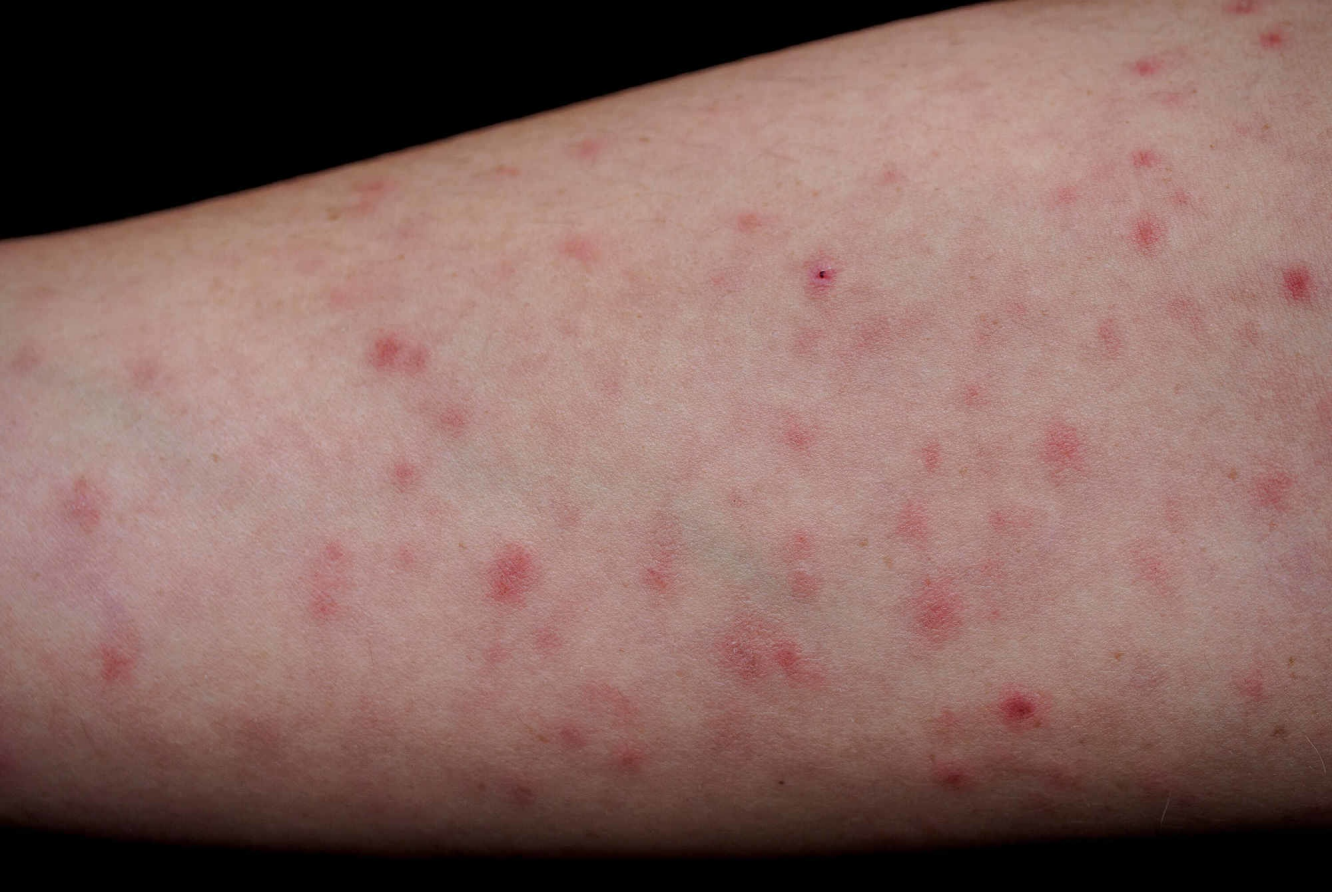
Red-purple-brown diffuse macules and papules with pigmented flecks distributed over the left volar forearm.

**Figure 2:**
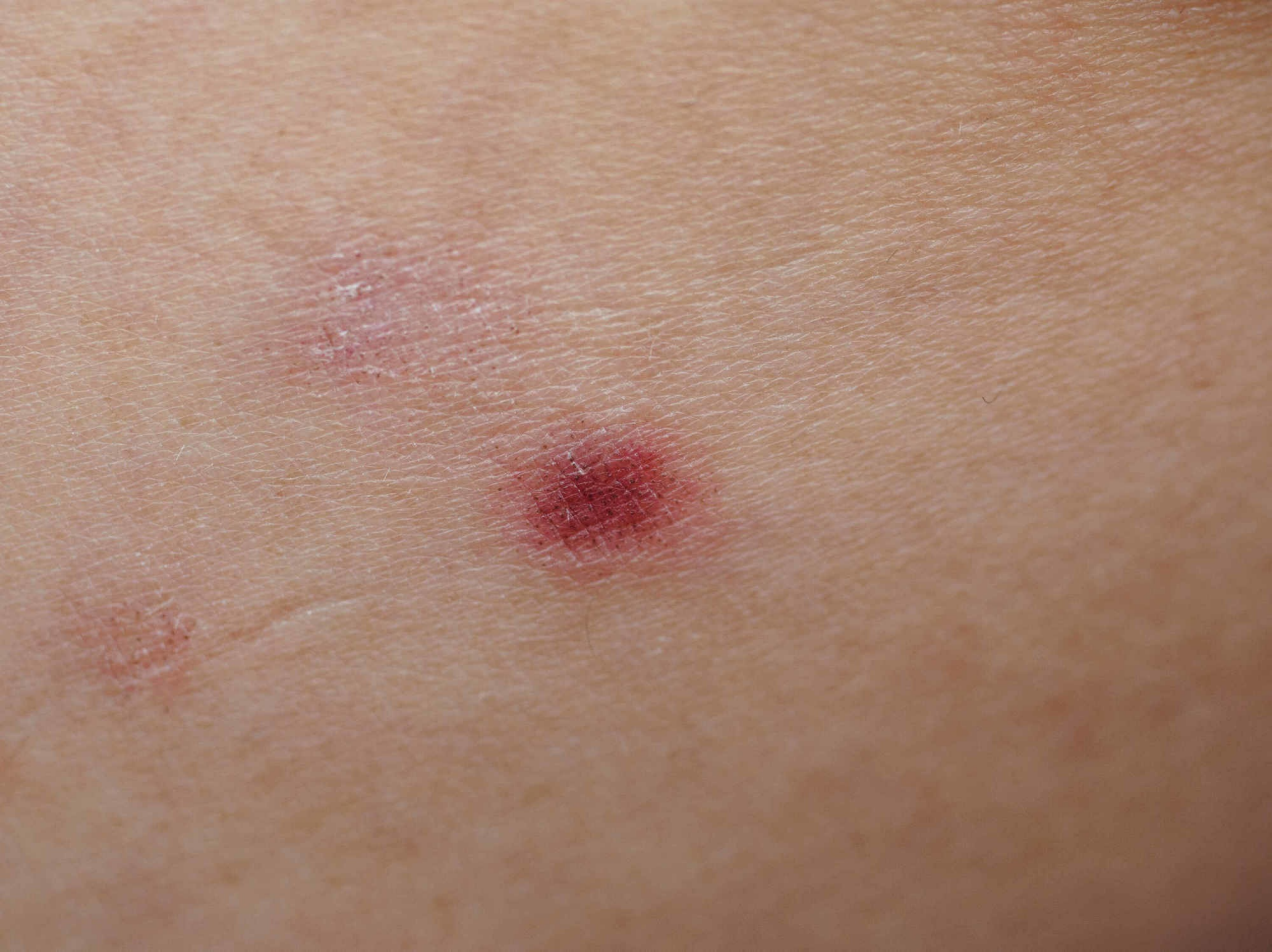
Close-up image of papule on the left volar forearm showing red thin papules with dark and light brown dots distributed within and outside of the red-purple coloration, respectively.

Upon seeing dermatology, there were varying red-purple-brown diffuse macules and papules with pigmented flecks on her bilateral arms (Figures 1, 2), trunk, and lower extremities.

**Figure 3:**
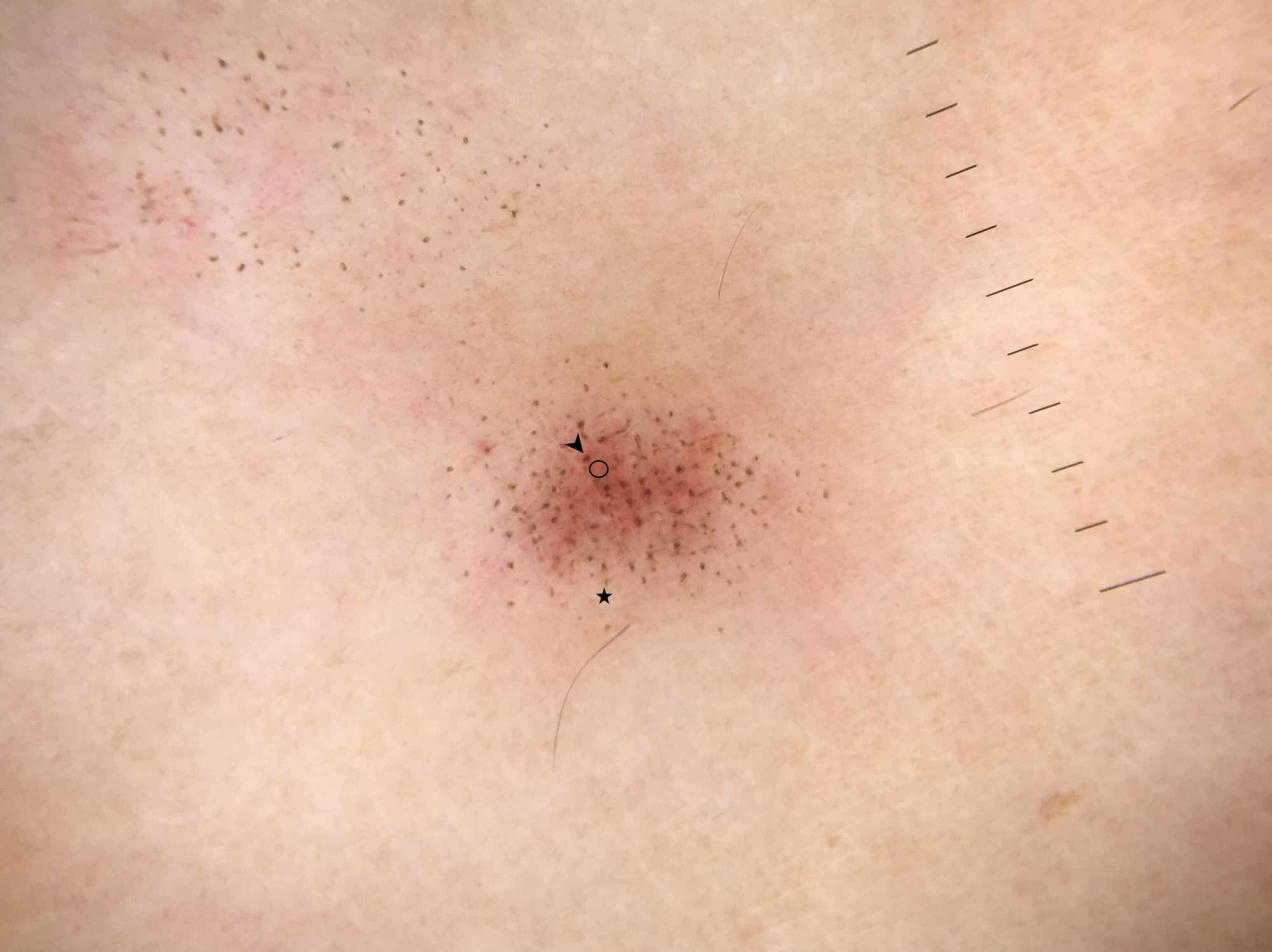
Dermoscopic view of papule in Figure 2 detailing pinpoint dark (black arrowhead) and light (black star) brown dots with underlying skin erythema (black circle) (×10, polarized; DermLite DL3N, 3Gen Inc, San Juan Capistrano, CA).

Under dermoscopy, a papule on the left volar forearm showed red thin papules with dark and light brown dots distributed within and outside of the red-purple coloration, respectively (Figure 3).

**Figure 4:**
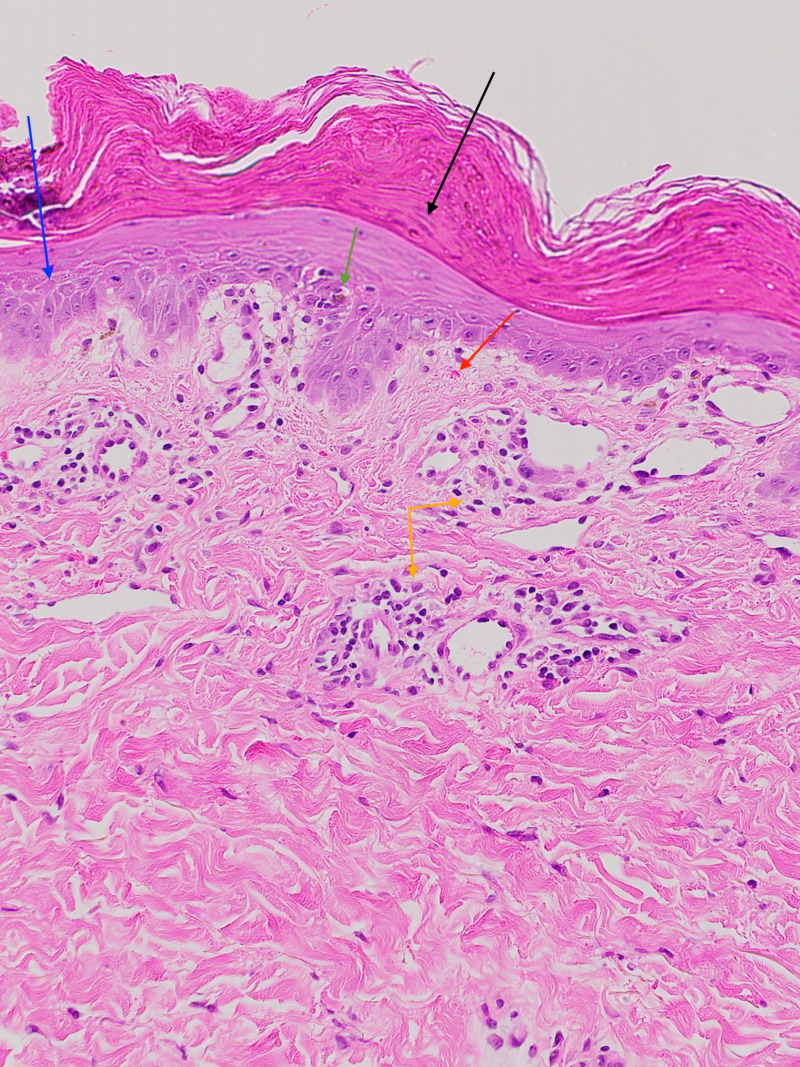
Focal parakeratosis (black arrow), mild spongiosis (blue arrow), mild superficial perivascular lymphocytic infiltrate (orange arrows), rare apoptotic keratinocytes (green arrow), and small numbers of extravasated erythrocytes (red arrow) (H&E, ×20).

Punch biopsy from a papule on the right proximal calf was obtained and demonstrated vacuolar interface alteration with a mild superficial perivascular lymphocytic infiltrate and small numbers of extravasated erythrocytes (Figure 4).

**Figure 5:**
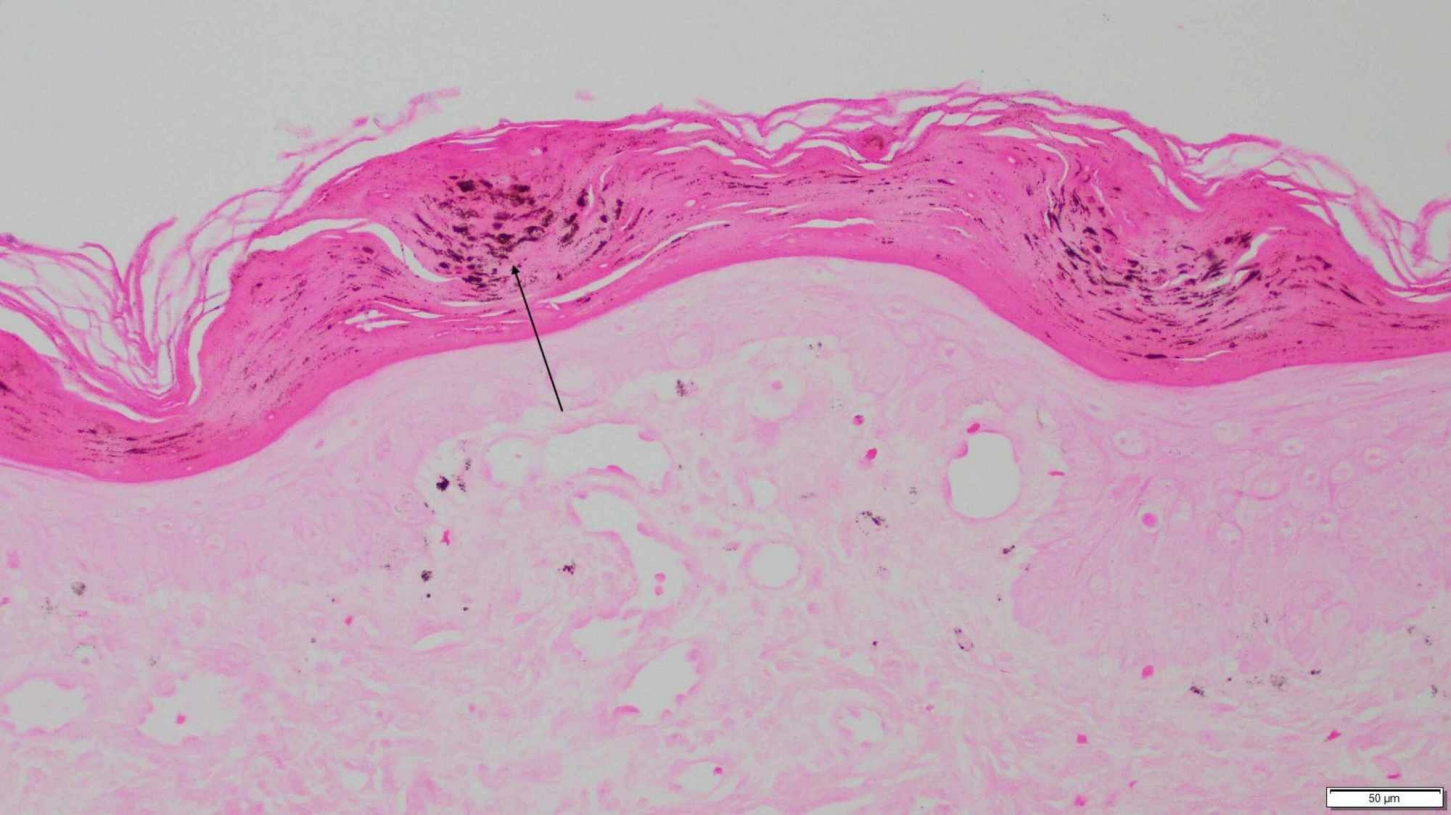
Focal melanin deposition within the stratum corneum (arrow) (Fontana-Masson, ×40).

Periodic acid-Schiff (PAS) staining was performed and was negative for fungus. Fontana-Masson staining was performed and was significant for focal melanin deposition within the stratum corneum (Figure 5). Iron staining was performed and was negative.

The findings were consistent with a clinical diagnosis of PLEVA. The patient was treated with doxycycline 100 mg twice daily. The option of phototherapy in the future was discussed. At one-month follow-up, significant improvement was noted with a near-complete response on the trunk and mild, red, thin papules on the arms. She was to continue doxycycline 100 mg twice daily with slow tapering off once no new lesions developed. 

## Discussion

Dermoscopy is most often utilized in the diagnosis of pigmented lesions. However, it has proven advantageous in the diagnosis of PLEVA due to its inexpensiveness, noninvasive technique, speed, and ability to differentiate from other diseases within the differential diagnosis [6]. A small number of reports have detailed PLEVA dermoscopy findings, with a 2010 report discussing individual cases of early and late phase lesions. The early phase lesion contained crusted brown and amorphous areas. The late phase lesion had a central white patch. In both cases, the lesions were surrounded by a ring of pinpoint/linear vascular structures with a targetoid aspect [3]. A 2016 study analyzed PLEVA in skin of color. Early phase lesions (n=2) were again significant for unstructured brown areas surrounding hair follicles with central scale and dark red dotted vessels on the periphery [4]. These dotted areas were thought to be secondary to microhemorrhages, red blood cell (RBC) extravasation in the dermis, and blood vessel dilatation. Late lesions differed by containing a variety of appearances, notably blue-gray areas (dermal melanin), and yellow globules (basal cell degeneration, spongiosis) [4].

In our report, dermoscopy revealed red thin papules with light and dark brown dots distributed outside and within of the discoloration, respectively (Figure 3). Histopathology was significant for focal positive staining within the stratum corneum on Fontana-Masson in the location of these spots, indicative of focal melanin deposition (Figure 5). No previous reports of PLEVA have described melanin deposition in the stratum corneum. However, PLC has been noted to have flecks of melanin within the stratum corneum with associated parakeratosis [8].

With this in mind, our case seems to fit better histologically with PLC but clinically with PLEVA, highlighting the spectrum of presentation in pityriasis lichenoides. The explanation for this stratum corneum melanin deposition, notably the focality of it, remains elusive, as one would expect dermal hyperpigmentation secondary to disruption of the basal layer with the lichenoid infiltrate and subsequent descent of melanin and melanophages into the dermis [9]. These findings may be nonspecific and may be secondary to dyskeratosis.

Histologic and dermatoscopic findings (based on case reports) are key in the differentiation of PLEVA from other diagnoses within the clinical differential diagnosis (Table 1). 

**Table 1:** Clinical differential diagnosis for PLEVA with associated dermatoscopic and histologic findings. MF, mycosis fungoides; ALCL, anaplastic large cell lymphoma; PLEVA, pityriasis lichenoides et varioliformis acuta.

Diagnosis	Clinical	Dermatoscopic	Histology
Lymphomatoid papulosis (LyP)	Papular, papulonecrotic, and/or nodular lesions, <1 cm, located most often on extremities and trunk. Recurrent crops, spontaneous regression weeks-months	Variable with clinical appearance and disease phase. Inflammatory papule: white center surrounding irregular, tortuous, radially arranged vessels [10]. Hyperkeratotic papule: enlargement of white center, less prominent vessels [10]. Papulonecrotic: prominent ulceration, loss of white structure [10]	Dermal infiltrate, often wedge-shaped and perivascular, of pleomorphic lymphoid cells resembling Reed-Sternberg cells (vesicular nucleus, prominent nucleoli with increased cytoplasm) [11]. Epidermal necrosis and ulceration possible [11]. Subtypes based on predominant cell type and tropism: A: CD30+ in large cells, most common (75%). B: decreased/absent CD30+; resembles MF. C: diffuse CD30+ cells; resembles ALCL. D: epidermotropic CD30+ cells; CD8+ predominates. E: angioinvasive CD30+ cells. F: folliculotropic CD30+ cells [12]
Viral exanthem	Varies depending on the cause, but most commonly presents as a widespread erythematous rash, often maculopapular, in association with systemic symptoms (fever, fatigue, headache)	Monomorphic papules with a central globule surrounded by an incomplete violaceous rim. Central purpuric globule in a background of erythema [13]	Sparse dyskeratotic keratinocytes in an otherwise unremarkable epidermis and a basket weave stratum corneum. Sparse lymphohistiocytic infiltrate
Cutaneous small vessel vasculitis	Most common cutaneous presentation is palpable purpura in dependent areas. Other findings include petechiae, ulcers, urticaria, hemorrhagic vesicles, and bullae	Blurry purpuric dots/globules (due to deep location of extravasated erythrocytes) and blue-gray patches [14]. Mottled purpura is suggestive of leukocytoclastic vasculitis [15]	Neutrophilic perivascular infiltrate. Erythrocyte extravasation. Fibrin deposition in the blood vessel wall. Karyorrhexis
Arthropod bite	Variety of causes, including mosquitoes, ants, fleas, bed bugs, ticks, and spiders. Presentation varies from erythematous papules to nodules, vesicles, and ulceration; punctum of the bite may be noted. Typically, self-limited	Variable depending on implicated arthropod, with case reports noting individual fleas, wasp stingers, and scabies eggs/mites [16]	Wedge-shaped mixed superficial inflammatory infiltrate (eosinophils, lymphocytes, histiocytes). Dermal edema. The extensive disease may note a deep lymphocytic infiltrate. Excoriation: parakeratosis, epidermal erosion, or ulceration
Guttate psoriasis	Scaly, water drop-sized (“gutta” = drop) plaques present over the trunk and extremities. Commonly noted in younger patients (<30). Group A streptococcal infection often precedes	Diffuse dotted vessels [6]	Epidermal hyperplasia, rete ridge elongation, dermal capillary dilatation and edema. Thin/absent granular layer. Neutrophils on mounds of parakeratosis
Varicella	Pruritic, erythematous maculopapular rash on the face, oral mucosa, trunk, and extremities, evolving to small vesicles on an erythematous base. Lesions oftentimes in different stages (vesicles, pustules, crusts)	White, ill-defined structures with surrounding erythema and peripheral brown dots [17]	Intraepidermal blister containing acantholysis and solitary keratinocytes. Epidermal multinucleated giant cells with nuclear molding

Without the utility of histologic and dermatoscopic findings illustrated in Table 1, several other important clues in the patient's presentation may assist in differentiating PLEVA from other diagnoses within its differential. For example, history including systemic symptoms (fever, fatigue, headache) may clue a provider towards a diagnosis of a viral exanthem. Organ-specific symptoms, including shortness of breath, cough, myalgias, and arthralgias, may raise clinical suspicion for vasculitis. Papulonecrotic or nodular lesions over the extremities and trunk raise clinical suspicion for lymphomatoid papulosis (LyP), notably important due to a roughly 15% risk of associated lymphoma (particularly mycosis fungoides [MF] and anaplastic large cell lymphoma [ALCL]) with LyP that is not present with PLEVA [18].

## Conclusions

PLEVA is a cutaneous disorder that is at times challenging to diagnose clinically. Our case is unique to the literature in that it lacks the ring of pinpoint vasculature, brown amorphous areas, and white scale seen in other reports, suggesting that there may be a wide range of dermoscopic findings for PLEVA present within an individual case. We presented the unique finding of focal melanin deposition within the stratum corneum. Further, we detailed the clinical differential diagnosis of PLEVA and noted both dermatoscopic and histopathologic findings of diagnoses within this differential. Additional reports illustrating dermatoscopic findings and histopathology for pityriasis lichenoides may be beneficial in increasing diagnostic confidence for this challenging diagnosis.
